# Association of 12-month contraceptive supply policy and months of oral contraception prescribed by obstetrics and gynecology resident physicians: an exploratory cross-sectional study

**DOI:** 10.1186/s12905-022-01869-w

**Published:** 2022-07-10

**Authors:** Megan F. Fuerst, Kaitlin Schrote, Bharti Garg, Maria I. Rodriguez

**Affiliations:** 1 Department of Obstetrics and Gynecology, Oregon Health & Science University, 3181 SW Sam Jackson Park Rd UHN 50, Portland, OR 97239 USA; 2 Center for Health Systems Effectiveness, Oregon Health & Science University, Portland, USA

**Keywords:** Contraception, Family planning, Medical education, Health policy

## Abstract

**Objective:**

This study sought to determine if there was a difference in the months of oral contraception prescribed by resident physicians living in U.S. states with a 12-month supply policy compared to resident physicians in states without a policy.

**Methods:**

We conducted an exploratory descriptive study using a convenience sample of Obstetrics and Gynecology resident physicians (n = 275) in the United States. Standard bivariate analyses were used to compare the difference between groups.

**Results:**

Few resident physicians in both groups (3.8% with a policy and 1.4% without a policy) routinely prescribed a 12-month supply of contraception. The mean coverage prescribed by providers in states with and without a policy was 2.81 and 2.07 months (*p* < 0.05).

**Conclusions:**

The majority of resident physicians were unaware of 12-month contraceptive supply policies and unable to correctly write a prescription for 12-months of contraception, regardless of whether they lived in a state with a 12-month contraceptive supply policy. Physician education may be needed to effectively implement 12-month contraceptive supply policies.

## Background

Access to contraception is an integral part of high-quality family planning care [1]. In the United States (U.S.), oral contraceptives (OCs) are the most common nonpermanent method of contraception [2]. To be effective, OCs should be taken continuously and with perfect use are 99% effective in preventing pregnancy [3]. Providing more pill packs at a single time makes it easier for individuals to take them continuously without unintended breaks [4–6].

Seventeen states have passed legislation requiring insurers to cover dispensation of a full 12-month supply of contraception [7]. The policy is designed to distribute 12 months of coverage, or 365 days of coverage, with a single refill at a single point in time. Such policies are linked to improved contraception continuation and reductions in unintended pregnancy as they reduce unnecessary trips to the pharmacy or office for refill requests that can cause disruptions in coverage [4, 6]. However, most individuals (70%) still receive less than a three-month supply of contraception at a time [5].

For these policies to be effective, insurance companies must comply with the law, and prescribers need to correctly write for a prescription to dispense 12-month supply of contraception. The objective of this exploratory study was to determine if there was a difference in the months of oral contraception prescribed by Obstetrics and Gynecology (OB/GYN) resident physicians living in U.S. states with 12-month supply policies as compared with physicians living in states without the policy. We hypothesized that resident physicians living in states with 12-month contraceptive supply policies would be more likely to prescribe higher amounts of OC at a single time.

## Methods

**Fig. 1:**
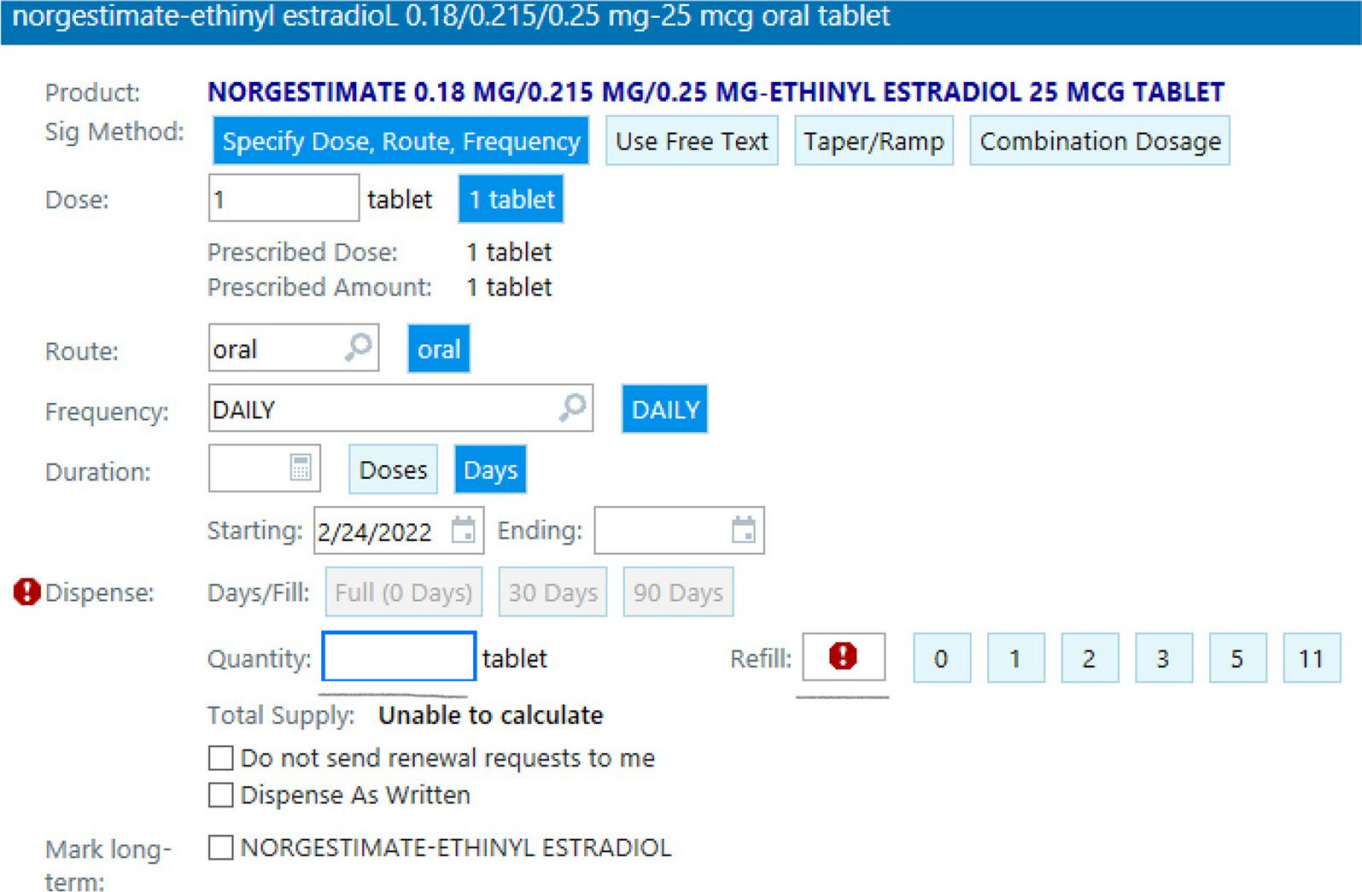
Sample survey question assessing resident physician ability to write a prescription for a 12-month supply of contraception

We conducted a cross-sectional survey of a convenience sample of OB/GYN residents and fellows in the U.S. The survey was titled “OB/GYN Trainees Attitudes on Pharmacist Prescribing Hormonal Contraceptives.” The survey was distributed electronically via educational listservs that included current U.S. OB/GYN residents and fellows. The survey took approximately ten minutes to complete, and participants were offered a ten-dollar Amazon gift card as incentive. Our primary outcome was the number of months of oral contraception prescribed. We measured this by having respondents fill in a prescription with their standard quantity of OCs prescribed and number of refills given (Fig. 1). To correctly have prescribed 12-months of contraception, respondents would have had to prescribe 12-months of OCs (ie: 365 tablets) with 1 refill. We also examined physician knowledge of the law and obtained demographic and practice characteristics of all respondents as well. The survey was available between November 4th and November 23, 2020, with a goal of achieving at least 250 responses. Of the states allowing for a 12-month contraceptive supply, the majority enacted their policies prior to 2020. South Carolina was the state to have most recently enacted a policy change (July 2021) and was therefore included as a state who had not yet adopted the policy. No states changed their contraceptive supply policy during the survey distribution period [7].

We compared responses by those residing in a state with a policy allowing for the provision a 12-month supply of contraception and those without the policy. Results were compared between the two groups using chi-squared test and Fisher’s exact test as appropriate. The study was approved by the Institutional Review Board at Oregon Health & Science University. Analyses were done using Stata/SE 15.1 (Stata Corp LP, College Station, TX, USA).

## Results

Our final sample size included 275 OB/GYN resident physicians from 27 states. Of the responses, 48% (n = 132) resided in a state with a policy requiring insurance coverage of a 12-month supply of contraceptives and 52% (n = 143) resided in states without such a policy. Relevant demographic factors are presented in Table 1. The majority of participants identified as White, Female, and were between the ages of 20 and 29. Participants in both groups reflected all levels of post-graduate training.

**Table 1:** Knowledge of 12-month contraceptive supply policy and demographic characteristics of obstetrician/gynecologist respondents. (n = 275)

	Resides in state without 12-month contraceptive supply policy (n, %) 143 (52)	Resides in state with 12-month contraceptive supply policy^a^ (n, %) 132 (48)	*p*-value
*Knowledge about contraception policy*			
Does your state have a policy requiring insurers to cover a 12-month supply of contraception?			0.49
Yes, and women are able to get a 12-month supply	27 (18.9)	35 (26.5)	
Yes, but only with certain insurance plans	9 (6.3)	7 (5.3)	
No	3 (2.1)	2 (1.5)	
I don’t know	104 (72.7)	88 (66.7)	
*Demographics*			
Race/ethnicity			0.49
White	99 (69.2)	80 (60.6)	
Black	6 (4.2)	9 (6.8)	
Hispanic	9 (6.3)	10 (7.6)	
Asian	23 (16.1)	24 (18.2)	
American native	1 (0.7)	2 (1.5)	
Age (years) *			0.003
20–29	86 (60.1)	65 (49.20)	
30–39	57 (39.9)	67 (50.80)	
Gender *			0.01
Female	126 (88.1)	116 (87.90)	
Male	16 (11.2)	15 (11.40)	
Gender variant, non-conforming	0	1 (0.8)	
Regions of the United States			0.47
Midwest	52 (36.4)	0 (0)	
Northeast	28 (19.6)	31 (23.5)	
South	42 (29.4)	8 (6.1)	
West	21 (14.7)	93 (70.5)	
*Program characteristics*			
Type of program*			< 0.001
Residency program	128 (89.5)	116 (87.9)	
Fellowship program	15 (10.5)	16 (12.1)	
Years in current training program*			< 0.001
1	35 (24.5)	37 (28.0)	
2	32 (22.4)	34 (25.8)	
3	31 (21.7)	31 (23.5)	
4	35 (24.5)	26 (19.7)	
5 +	10 (7.0)	4 (3.0)	

^a^States with policy at time of survey distribution include California, Colorado, Connecticut, D.C., Delaware, Hawaii, Maine, Maryland, Massachusetts, Nevada, New Hampshire, New Jersey, New Mexico, Oregon, Rhode Island, South Carolina, Vermont, Virginia, Washington

*p < 0.05 for Chi-squared or Fisher’s exact test

Overall, knowledge of the policy and months of contraception prescribed was low in both groups. In states with a 12-month contraceptive supply policy, 66.7% of respondents did not know such a policy existed. Similarly, in states without such a policy, 72.7% responded that they did not know whether their state of practice had such a policy.

When asked to write a prescription for a 12-month supply of contraception, respondents on average wrote a prescription for 2.40 (Standard Deviation, SD = 1.85) months of OCs. The mean months of contraception dispensed by respondents in states with and without a policy were 2.81 (Standard Deviation, SD 2.07) and 2.02 (SD 1.54), respectively (*p* < 0.05). Both groups routinely prescribed a median of 4 refills. Only 6.10% of respondents in states with a 12-month supply policy did this correctly in comparison to 2.80% percent of respondents in states without a 12-month supply policy (*p* = 0.19). There was no association with the year of training and the amount of contraception prescribed (*p* = 0.80).

**Fig. 2:**
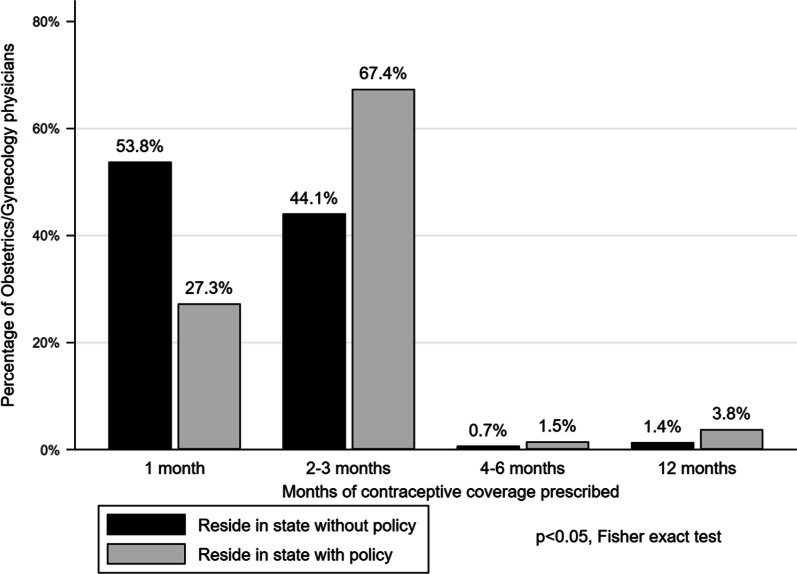
Months of contraceptive coverage prescribed by obstetrics & gynecology physicians living in states with and without a policy requiring insurance coverage of 12-months of contraception (n = 275)

Similarly, in states with a 12-month contraceptive policy in place, 3.8% of resident physicians reported regularly prescribing a 12-month supply, while only 1.4% of physicians in states without a policy reported regularly prescribing a 12-month supply (*p* < 0.05 for Fisher’s Exact Test, Fig. 2). The majority of respondents in states without a policy, 53.8%, prescribed 1-month of coverage at a time; while the majority of respondents in states with a contraceptive supply policy prescribed between 2 and 3 months of coverage at a time (*p* < 0.05 for Fisher’s Exact Test, Fig. 2).

## Discussion

For 12-month contraceptive supply policies to be fully implemented, prescribers must be aware of the law and insurance companies held accountable to fulfilling the legislation. Not all states have applied the policy to national insurers such as Medicaid and some insurers may still place limits on coverage such as requiring the insured person to first receive a limited supply of contraception. Moreover, private companies who are based out of state would not be bound by the state-level law [7]. Resident physicians in states with the policy were slightly more likely to prescribe a 12-month supply. However, the proportion of physicians aware of the policy and prescribing a 12-month supply were very low in both groups, and unlikely to impact population health.

Health policy is a key determinant of care; however, policy implementation requires engagement with all affected stakeholders. Adequately training healthcare providers is relatively low-hanging fruit when it comes to effectively implementing health policy [8]. The results of this study suggest that provider-level trainings may have a large impact on the currently low percentage of physicians prescribing a 12-month supply of contraception. This would improve oral contraception access for birthing individuals in the seventeen states with a policy already in place. As U.S. OB/GYN residency programs are required to offer regular didactic sessions, the population used for this study would be an easy target to pilot such trainings. This could then be expanded to other medical specialties or advanced healthcare practitioners who also prescribe contraception. Providers in states without a policy could still benefit from learning about the policy to better advocate for patients at a state-level.

To further improve implementation of 12-month contraceptive supply policies, future studies can focus on other barriers to equitable implementation. On the provider side, further investigations can explore other factors that impact provider prescribing, such as default electronic medical record prescription settings, institutional policies, and implicit bias [9, 10]. Pharmacies must also be comfortable prescribing a 365-day supply and have adequate supply in stock to dispense. Regardless, the low proportion of providers in this study able to identify 12-month contraceptive supply policies, let alone correctly prescribe a 12-month supply of contraception requires attention if the current 12-month contraceptive supply policies are to impact contraception access.

This study is limited in that it was a relatively small, cross-sectional study. We did not assess other barriers to implementing 12-month contraception supply policies, such as insurance company coverage and patient preferences [4, 6]. Additionally, we only surveyed resident physicians and our study population may not represent the entire healthcare workforce who prescribes contraception. However, there are over 100,000 resident physicians employed at any given time and they make up a key component of the current and future physician workforce [11]. Based on the convenience sampling used for survey distribution, we cannot calculate the exact survey response rate. However, assuming that there are approximately 1400 OB/GYN resident physicians in the U.S., the survey response rate is likely at least twenty percent. Additionally, the average number of months of OC prescribed by respondents in this survey (2.40 months of coverage), is similar to other studies showing that on average between two and four months of OC coverage are most frequently dispensed at a single time [5, 12]. The policy applies to all short acting forms of contraception, including the contraceptive patch and the contraceptive ring. We chose to only focus on OCs in this study given that they are the most common form of short-acting contraception [2]. The survey did not ask questions about resident physician experience and training in contraception prescribing. Future studies should evaluate the impact resident education can have on prescribing as well as other barriers to accessing a 12-month supply of contraception, including pharmacist and insurance level barriers.

## Conclusions

The results of this exploratory study suggest that OB/GYN providers are largely unaware of 12-month contraceptive dispensing policies and how to correctly prescribe a 12-months of contraception. Provider training on this topic could be helpful to increase knowledge in this population and improve policy implementation. Additional research should examine other barriers to policy implementation.

## Data Availability

The datasets used and/or analysed during the current study are available from the corresponding author on reasonable request.
